# SYNE1 mutation may enhance the response to immune checkpoint blockade therapy in clear cell renal cell carcinoma patients

**DOI:** 10.18632/aging.103781

**Published:** 2020-10-08

**Authors:** Pengju Li, Jeifei Xiao, Bangfen Zhou, Jinhuan Wei, Junhang Luo, Wei Chen

**Affiliations:** 1 Department of Urology, The First Affiliated Hospital, Sun Yat-Sen University, Guangzhou, Guangdong Province, P. R. China; 2 Department of Extracorporeal Circulation, The First Affiliated Hospital, Sun Yat-Sen University, Guangzhou, Guangdong Province, P. R. China; 3 Department of Urology, The First Affiliated Hospital of Hainan Medical University, Haikou, Hainan, P.R.China

**Keywords:** SYNE1 mutation, tumor mutation burden, immune response, immune cells infiltration

## Abstract

As one of the 10 most common cancers in men, the incidence of renal cell carcinoma (RCC) has been increasing in recent years. Clear cell renal cell carcinoma (ccRCC) is the most common pathological type of RCC, counting for 80%-90% of cases. Immunotherapy is becoming increasingly important in the treatment of advanced RCC. Tumor mutation burden (TMB) is a potent marker for predicting the response to immune checkpoint blockade (ICB) treatment. Here, we analyzed somatic mutation data for ccRCC from The Cancer Genome Atlas datasets. We found that the frequently mutated gene SYNE1 is associated with higher TMBs and with a poor clinical prognosis. To further investigate the relationship between SYNE1 mutation and the immune system, we used Gene Set Enrichment Analysis and the CIBERSORT algorithm. They showed that SYNE1 mutations correlate with immune system pathways and immune cell tumor infiltration. We also found that SYNE1 mutation correlated with a better response to ICB therapy. Thus, mutation of SYNE1 correlates with a higher TMB and a poorer outcome in ccRCC, but may mediate better responses to ICB therapy.

## INTRODUCTION

Renal cell carcinoma (RCC) is a common malignant tumor of the urinary system, the incidence of which has been increasing. It is estimated that there will be 73,750 new cases of RCC in 2020 and 14,830 patients will die of the disease [1]. Clear cell renal cell carcinoma (ccRCC) is the most common pathological type of RCC, accounting for 80%-90% of cases [1, 2]. The clinical symptoms of early RCC are not obvious, and distant metastasis has occurred in 20% -30% of cases by the time of diagnosis. RCC is not sensitive to conventional radiotherapy or chemotherapy, which are effective in less than 20% of cases. Consequently, the prognosis of RCC with metastasis is poor, with a median survival time of only about 10 months [3].

Therapy targeting vascular endothelial growth factor and the rapamycin target protein pathway is effective, but drug resistance is nearly inevitable [4]. Recently, there has been significant progress in treating metastatic RCC with immunotherapy. In contrast to nonspecific immunotherapies with IL-2 or IL-6, immune checkpoint blockade (ICB) therapy has shown substantial efficacy against advanced RCC since being approved as a second-line treatment in 2015. ICB therapy based on programmed death 1 (PD1)/programmed death ligand (PDL1) and cytotoxic T lymphocyte associated antigen (CTLA4) has shown significant survival benefits in many cases of advanced RCC [5–8]. However, only about 20% of patients benefit from the therapy [9], and it is necessary to screen patients using predictive biomarkers to determine the potential efficacy of ICB therapy. In that regard, it is has been shown that tumor mutation burden (TMB) is associated with the response to immunotherapy [10], and that accumulation of somatic mutations correlates with neoantigen expression [11]. Thus, TMB [12], PDL1 [13], and tumor-infiltrating lymphocytes (TILs) [14] have been identified as biomarkers in various solid tumors.

The frequently mutated gene, SYNE1, encodes a series of spectrin structural proteins, which play key roles in cytoskeletal, nuclear and vesicular anchoring [15], and its mutation is associated with a form of cerebellar ataxia [16]. In addition, recent evidence suggests changes in SYNE1 expression levels, somatic mutations, and single nucleotide polymorphisms are related to the occurrence and development of lung cancer [17], oral cancer [18], hepatocellular carcinoma [19], and gastric cancer [20]. In the present study, we analyzed datasets of somatic mutations from The Cancer Genome Atlas (TCGA) in ccRCC patients. In the present study, we assessed the relation between SYNE1 and TMB and prognosis. We also compared immune cell infiltration between those with SYNE1 mutation type (mt) and those with wild type (wt), and evaluated the utility of SYNE1 mutation as a ICB biomarker using the tumor immune dysfunction and exclusion (TIDE) algorithm [21]. Our results suggest SYNE1 may be useful for predicting the efficacy ICB therapy.

## RESULTS

### Somatic mutations, TMB and clinical outcomes in ccRCC patients

**Figure 1:**
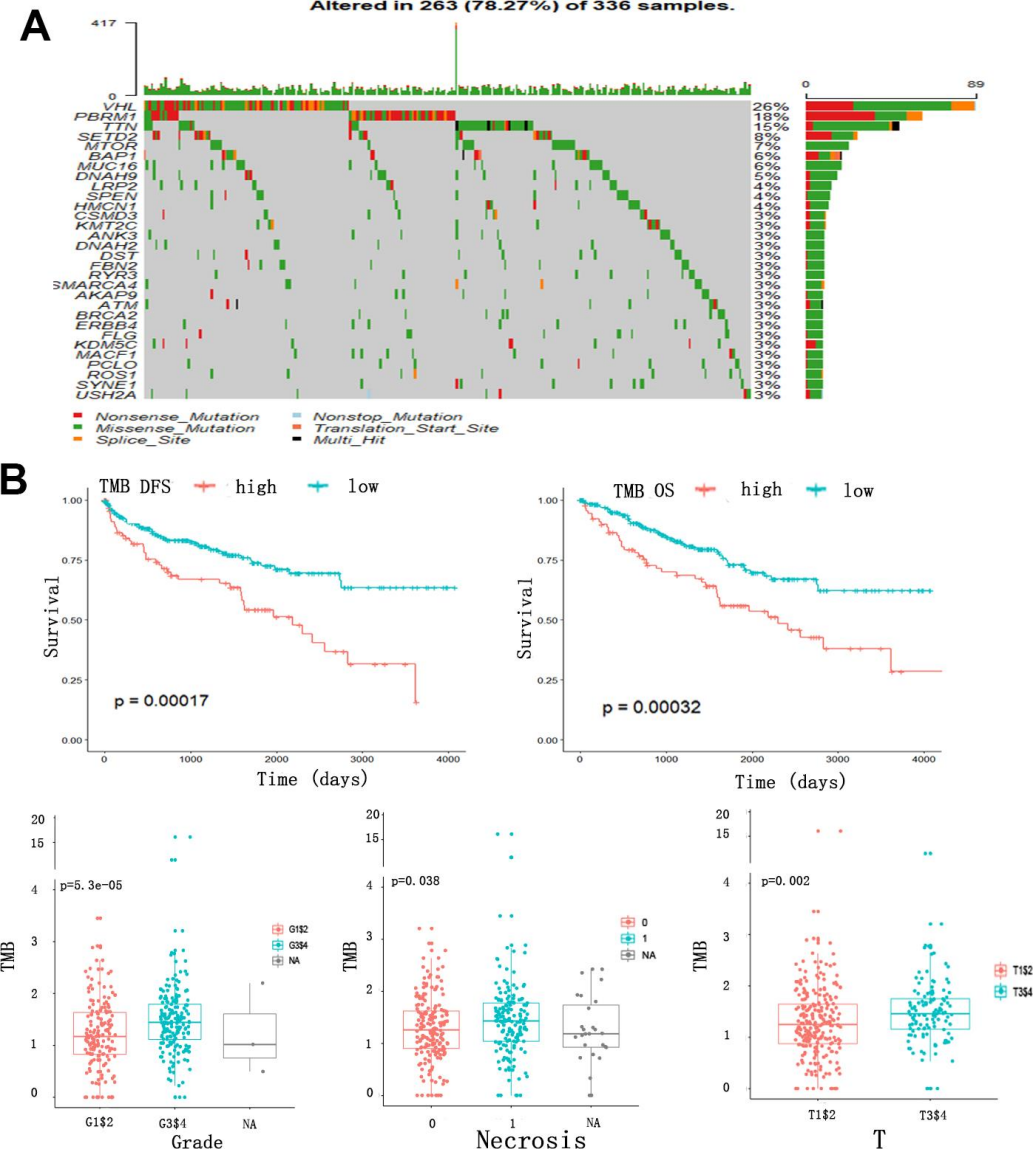
**Somatic mutation, TMB and clinical outcomes in ccRCC patients.** (**A**) Oncoplot for frequently mutated genes in ccRCC samples from TCGA cohort. Genes are listed by mutation frequency. The bottom panel shows the different mutation types. (**B**) TMB and clinical outcomes in ccRCC patients. X-tile plot of TMB and OS. A TMB score cutoff of 1.7 was used to divided patients into TMB-low and TMB-high subsets.

We analyzed the somatic mutations in ccRCC patients from TCGA cohort and identified 30 frequently mutated genes (Figure 1A). The genes and percentages of patients carrying the mutations are as follows: VHL (26%), PBRM1 (18%), TTN (15%), SETD2 (8%), MTOR (7%), BAP1 (6%), MUC16 (6%), DNAH9 (5%), LRP2 (4%), SPEN (4%), HMCN1 (4%), CSMD3 (3%), KMT2C (3%), ANK3 (3%), DNAH2 (3%), DST(3%), FBN2 (3%), RYR3 (3%), MARCA4 (3%), AKAP9 (3%), ATM (3%), BRCA2 (3%), ERBB4 (3%), FLG (3%), KDM5C (3%), MACF1 (3%), PCLO (3%), ROS1 (3%), SYNE1 (3%), USH2A (3%). We also evaluated the correlation between TMB and clinical outcomes using data collected from Cbioportal datasets. The TMB scores across all samples ranged from 0 to 16.05157. After constructing an X-tile plot of TMB and overall survival (OS), a TMB score cutoff of 1.7 was used to divide patients into TMB-low and TMB-high subsets. In contrast to findings from earlier studies of other cancers [22, 23], ccRCC patients in TMB-high had poorer clinical outcomes, irrespective of their disease-free survival (DFS) (p=0.00017) or OS (p=0.00032) status. At the same time, a higher TMB indicated a higher tumor grade (p<0.0001), higher risk of necrosis (p=0.038), and higher T staging (p=0.002) (Figure 1B).

### TMB and survival prognosis based on SYNE1 mutation and enrichment pathway analysis of SYNE1 mutation

**Figure 2:**
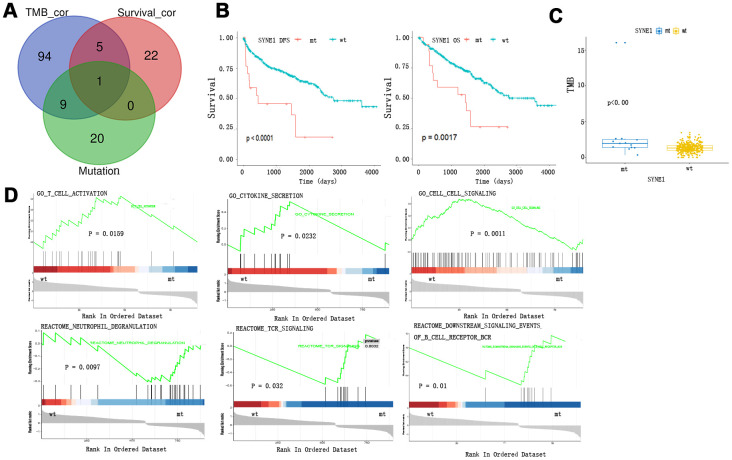
**TMB and survival prognosis based on SYNE1 mutation and enrichment pathway analysis.** (**A**) Venn diagram of frequently mutated genes showing TMB correlated and survival correlated mutated genes. (**B**) SYNE1 mutation and survival prognosis. (**C**) SYNE1 mutation is related to a higher TMB. (**D**) GSEA enrichment based on SYNE1 mutation: wt, wild type; mt, mutant type.

We detected the SYNE1 mutation in a Venn diagram at the intersection of the top 30 somatic mutations, survival-related mutations, and TMB-related mutations in ccRCC (Figure 2A). Patients with SYNE1 mutation (mt) had poorer survival outcomes, irrespective of DFS (p<0.0001) or OS (p=0.0017) status (Figure 2B). In addition, a higher TMB correlated with SYNE1 mutation (p<0.0001) (Figure 2C). This is consistent with our previous analysis of TMB and clinical outcomes. COX survival analysis revealed that SYNE1 mutation was a risk factor affecting prognosis (HR=0.978; 95% CI, 1.156-6.114; P=0.021). In a multivariate analysis, however, SYNE1 mutation did not remain a significant factor affecting prognosis (Table 1), suggesting SYNE1 mutation may not be an independent risk factor affecting prognosis in ccRCC patients. GSEA results revealed that pathways including GO T cell activation, GO cytokine secretion, GO cell-cell signaling, Reactome neutrophil degranulation, Reactome TCR signaling, and Reactome downstream signaling events of B cell receptor are involved (Figure 2D).

**Table 1:** Univariate and multivariate overall survival analysis of ccRCC patients by the COX proportional hazards model.

**Factors**	**Univariate**		**Multivariate**	
	**HR(95%CI)**	**p value**	**HR(95%CI)**	**p value**
Grade(G1$2,G3$4)	-0.839 (0.268-0.698)	0.001		
Stage(stage1$2,stage3$4)	-1.435 (0.150-0.378)	0.000	-1.478 (0.144-0.362)	0.000
TMB(high,low)	0.673 (1.401-2.743)	0.000		
SYNE1(mt,wt)	0.978 (1.156-6.114)	0.021		
Gender(male,female)	0.343 (0.918-2.161)	0.117		
Age(<70y,>70y)	1.015 (1.774-4.292)	0.000	1.087 (1.902-4.621)	0.000

### Tumor-infiltrating immune cells associated with SYNE1 mutation in ccRCC

To further compare the differential profiles of immune fractions between SYNE1 mt and wt groups, we used the CIBERSORT algorithm to evaluate the association between SYNE1 mutation and tumor-infiltrating immune cells. Twenty-two immune cell types in each ccRCC sample are shown in the boxplot in Figure 3A. We found that numbers of CD8 T cells (p=0.015), monocytes (p=0.046), resting dendritic cells (p=0.031), and eosinophils (p=0.024) were lower in the SYNE1 mt group than in the wt group (Figure 3B). Using a correlation matrix, we found that numbers of CD8 T cells correlated positively with follicular helper T (TH) cells, regulatory T cells (Tregs) and M1 macrophages, while they correlated negatively with CD4 T cells, resting memory T cells, and M0 macrophages (Figure 3C). The heatmap shows that immune cell fractions were lower in the mt group than wt group (Figure 3D).

**Figure 3:**
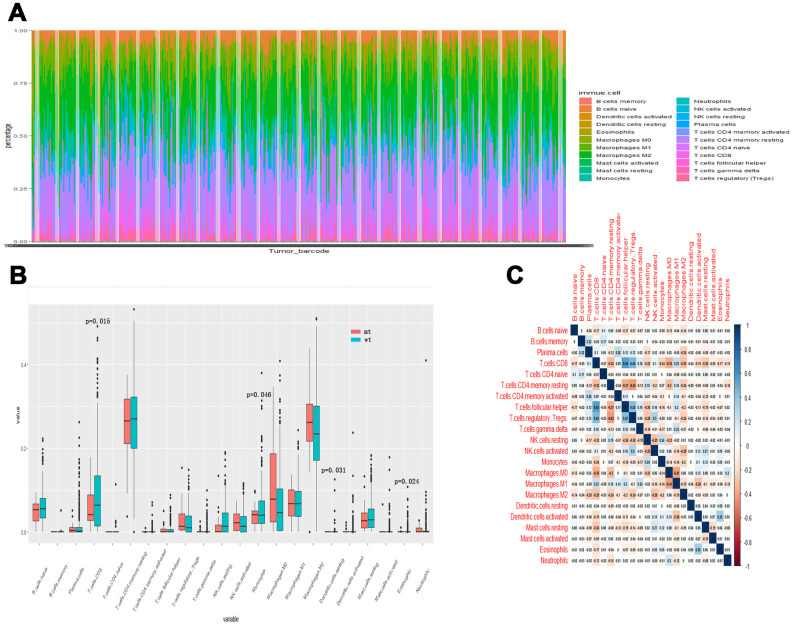
**Tumor-infiltrating immune cells associated with SYNE1 mutation in ccRCC.** (**A**) Bar chart of infiltration of 22 immune cells. (**B**) Boxplot showing differentially infiltrating immune cells based on SYNE1 mutation. Red color represents the mt group and blue represents the wt group. (**C**) Correlation matrix of immune cell fractions. The blue color represents positive correlation, and red represents negative correlation.

**Figure 3:**
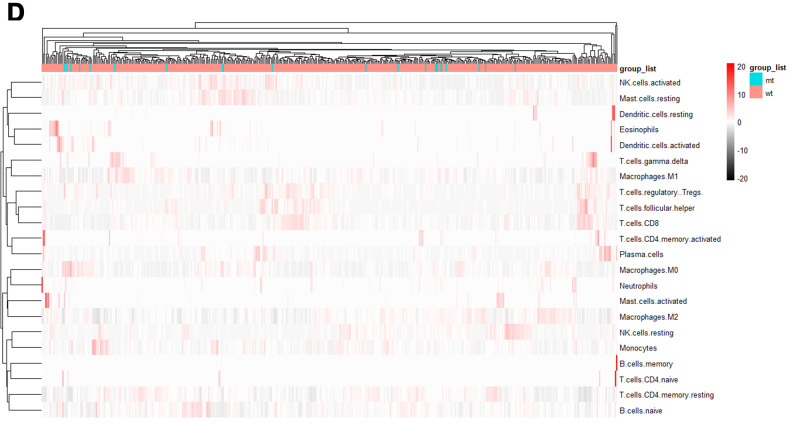
**Tumor-infiltrating immune cells associated with SYNE1 mutation in ccRCC.** (**D**) Heatmap of 22 immune cell types based on SYNE1 mutation. The blue color represents the mt group, and red represents the wt group.

### Predicting of ICB response based on SYNE1 mutation and biomarker evaluation

**Figure 4:**
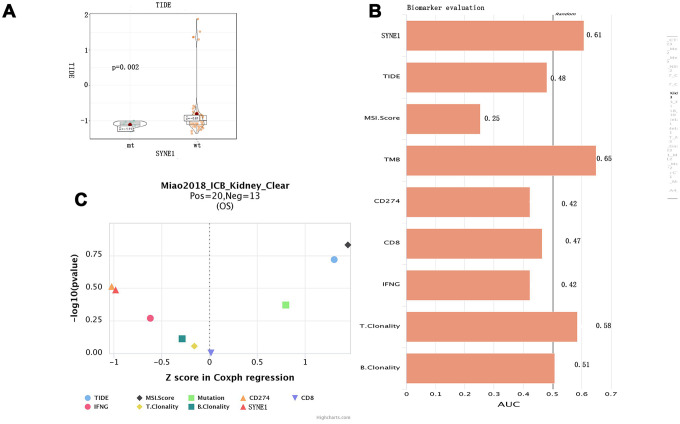
**Predicting of ICB response based on SYNE1 mutation and biomarker evaluation.** (**A**) Violin plot showing the differential TIDE between the mt and wt groups. (**B**) Evaluation of SYNE1 as a biomarker compared with existing biomarkers. (**C**) Multivariate analysis of SYNE1 and existing biomarkers using the COX proportional hazards model.

To evaluate the ability of SYNE1 mutation to served as a biomarker predictive of the clinical response to ICB therapy, we analyzed datasets from patients with stage 4 ccRCC using the TIDE algorithm. The results showed that the mt group was associated with lower TIDE scores, indicating a stronger response to ICB therapy (Figure 4A). To evaluate its utility as a biomarker, we compared SYNE1 mutation to other existing biomarkers. Bar plots showed that SYNE1 has good prediction accuracy (Figure 4B). Lastly, our results suggest that SYEN1 mutation may be an independent risk factor associated with prognosis for ICB therapy (Figure 4C).

## DISCUSSION

In recent years immunotherapy used to treat ccRCC patients has changed from traditional nonspecific therapy to specific immunotherapy based on ICB. This greatly improved the efficacy of treatment for advanced RCC. Unfortunately, most RCC patients do not exhibit an immune response with ICB; only a small percentage of patients obtain a benefit. Consequently, it is necessary to identify patients who are more likely to respond to ICB therapy before administering the drug.

In the present study, we analyzed TCGA datasets of somatic mutations in ccRCC patients. We found that SYNE1 was frequently mutated in TCGA cohorts. In contrast to outcomes in patients with other tumors, where higher TMBs correlated with more favorable clinical prognoses, SYNE1 mutation was reportedly associated with higher TMB and poorer clinical prognoses [24]. Our findings are consistent with that earlier observation. GSEA enrichment analysis showed that SYNE1 mutation correlates negatively with T cell activation and cytokine secretion, and correlates positively with neutrophil degranulation and T cell receptor (TCR) signaling. The role of neutrophils in cancer is complex. On the one hand, they can kill tumor cells, but on the other hand, neutrophil degranulation can promote tumor cell immune escape and metastasis [25]. Activation of TCR signaling is closely related to T cell activity, leading to creation of new epitopes. It is reported, however, that only 0.3%-1.3% of tumor gene mutations induce T cell responses via TCR signaling [26]. Firstly, the rate of nonsynonymous mutations and frameshifts occur in protein-coding sequences is low. Secondly, proteasome cleavage of mutation-containing protein can destroy TCR-recognized peptide epitopes [27].

Samples with SYNE1 mutations were correlated with signaling pathways involved in immune responses and alterations in the profiles of infiltrating immune cells. For example, CD8 T cell and monocyte fractions were significantly lower in the mt than wt group. Moreover, recent studies have shown that lower CD8 T cell and monocyte infiltration is closely related to a poor prognosis [28, 29]. Using a correlation matrix, we could see CD8 T cells correlated positively with TH cells and Tregs. There are two parts to the T cell activation signal pathway. The first entails activation TCR signaling; the second involves the assistance of molecules such as CD8 and CD4, which are expressed in TH cells and Tregs [30]. This may explain the results of our GSEA enrichment pathways analysis, which indicated that SYNE1 mutations correlate positively with TCR signaling but correlate negatively with T cell activation.

We evaluated the ability of SYNE1 mutation to serve as a biomarker. Interestingly, although we found that the mt group showed higher TMBs and poorer prognoses, it also correlated with a better response to ICB therapy. And compared with existing biomarkers, SYNE1 mutation exhibited good prediction ability. These findings suggest SYNE1 mutations may play a role in ICB therapy in ccRCC.

Our study has several limitations. First, the sample size was relatively small, and a clinical trial with a larger sample size is needed to validate our hypothesis. In addition, basic experiments should be performed to verify and clarify the mechanism.

## MATERIALS AND METHODS

### Clinical cohorts and the mutation data

In this study, we analyzed the relationship between TMB and clinical outcome in 443 patients from the cBioPortal database (https://www.cbioportal.org) and the supplemental file, “mutation-load-updated.txt,” of a TCGA Pan-Cancer study (https://api.gdc.cancer.gov/data/ff3f962c-3573-44ae-a8f4-e5ac0aea64b6) [31]. TMB was determined as the total number of mutations per sample, normalized by the whole-exome sequencing coverage, as described in Knijnenburg et al [32]. Intronic mutations, mutations in the 3′ or 5′ UTR regions or UTR flanking regions, silent mutations, and small, in-frame insertions and deletions were all removed. TCGA clinical data were download from TCGA Pan-Cancer Clinical Data Resource (TCGA-CDR) (https://ars.els-cdn.com/content/image/1-s2.0-S0092867418302290-mmc1.xlsx). SYNE1 mutation data were download from the Kidney Renal Clear Cell Carcinoma (TCGA, Firehose Legacy) dataset (http://download.cbioportal.org/kirc_tcga.tar.gz).

### Bioinformatic analysis

MAF files containing somatic variants for American ccRCC samples were download from TCGA datasets and visualized using the maftools package [33]. Gene set enrichment analysis (GSEA) was performed with R studio script [34]. Gene expression data were downloaded from TCGA and divided into two groups based on SYNE1 mutation status. The gene set “msigdb.v7.0.entrez.gmt” was downloaded from the Molecular Signatures Database (http://software.broadinstitute.org/gsea/msigdb/index.jsp) and was used for the enrichment analysis. The CIBERSORT algorithm was used to evaluate the fractions of 22 tumor-infiltrating lymphocyte subsets and were compared based on SYNE1 mutation status [35]. The TIDE algorithm was used to predict ICB responses and evaluate ability to serve as a neoantigen (http://tide.dfci.harvard.edu) [21]. Values of p > 0.05 were considered statistically significant.

### Statistical analyses

X-tile plots provided a single and intuitive method to assess the association between TMB and patient survival [36]. The Kaplan-Meier method was used to analyze the correlation between SYNE1 mutation, TMB and patient survival, and the log-rank test was used to compare survival curves. Statistical tests were performed using R software, version 3.6.2 (R Foundation for Statistical Computing; Vienna, Austria). Univariate and multivariate Cox regression analyzing the association between clinical characteristics and survival was performed using SPSS, version 19. Values of p > 0.05 were considered statistically significant.
